# Effects of home-based play-assisted stimulation on developmental performances of children living in extreme poverty: a randomized single-blind controlled trial

**DOI:** 10.1186/s12887-018-1023-0

**Published:** 2018-02-05

**Authors:** Berhanu Nigussie Worku, Teklu Gemechu Abessa, Mekitie Wondafrash, Johan Lemmens, Jan Valy, Liesbeth Bruckers, Patrick Kolsteren, Marita Granitzer

**Affiliations:** 1 Department of Psychology, Jimma University, Jimma, Ethiopia; 2 Department of Special Needs and Inclusive Education, Jimma University, Jimma, Ethiopia; 3 Department of Population and Family Health, Jimma University, Jimma, Ethiopia; 4 Department of Healthcare, PXL University College, Hasselt, Belgium; 5 Interuniversity Institute for Biostatistics and Statistical Bioinformatics, Hasselt University, Hasselt, Belgium; 6 Department of Food Safety and Food Quality, Faculty of Bioscience Engineering, Ghent University, Ghent, Belgium; 7 REVAL Rehabilitation Research Centre, Biomedical Research Institute, Faculty of Medicine & Life Sciences, Hasselt University, Hasselt, Belgium

**Keywords:** Developmental performance, Developmental stimulation, Extreme poverty, Foster family, Home-based, Play-assisted stimulation

## Abstract

**Background:**

Children living with foster families in a resource-limited setting such as Ethiopia are at risk of developmental problems. It is not yet clear whether intensive home-based developmental stimulation assisted by play can reduce these problems. The main objective of this study was to examine the effects of play-assisted intervention integrated into basic services on the developmental performance of children living with foster families in extreme poverty.

**Methods:**

A randomized single-blind (investigator) controlled trial design was used. The study was conducted in Jimma, South West Ethiopia. Using computer-generated codes, eligible children of 3–59 months in age were randomly allocated to intervention (*n* = 39) and control (*n* = 39) groups at a 1:1 ratio. Children in the intervention group received home-based play-assisted stimulation in addition to the basic services provided to children in both groups. The intervention consisted of an hour of play stimulation conducted during a weekly home visit over the course of six months. Personal-social, language, fine and gross motor outcomes were assessed using Denver II-Jimma, and social-emotional outcome was obtained using an adapted Ages and Stages Questionnaire: Social-Emotional (ASQ: SE). Information about sociodemographic characteristics was collected using a structured questionnaire. Anthropometric methods were used to determine nutritional status. The effects of the intervention on the abovementioned outcomes over the study period and group differences in change over time were examined using Generalized Estimating Equations (GEE).

**Results:**

Statistically significant intervention effects were found for language (*P* = 0.0014), personal-social (*P* = 0.0087) and social-emotional (*P* <  0.0001) performances. At the midline of the study, language (effect size = 0.34) and social-emotional (effect size = − 0.603) benefits from the play-assisted stimulation had already been observed for the children in the intervention group. For language, the intervention effect depended on the child’s sex (*P* = 0.0100) and for personal-social performance, on family income (*P* = 0.0300).

**Conclusions:**

Intensive home-based play-assisted stimulation reduced the developmental problems of children in foster families in the context of extreme poverty. Longer follow-up may reveal further improvements in the developmental performance of the children.

**Trial registration:**

The study was retrospectively registered on ClinicalTrials.gov on 17 November 2016, Study Identifier: NCT02988180.

**Electronic supplementary material:**

The online version of this article (10.1186/s12887-018-1023-0) contains supplementary material, which is available to authorized users.

## Background

Child poverty is particularly critical in Sub-Saharan Africa, and half of the world’s extremely poor children currently live in this region. Most of these children are at risk of health issues, as well as developmental problems [1, 2]. The main reason for this, is that extreme poverty is strongly linked to undernutrition, poor sanitation, poor maternal education, increased maternal stress and depression, as well as restricted learning opportunities and inadequate stimulation at home [3–5]. These factors are rooted in absolute poverty and food insecurity, and together they negatively affect child development [6, 7]. Childhood undernutrition, for example, is intensely embedded in poverty [8], and detrimentally affects child development [7]. Maternal mental health can affect the quality of mother-child attachment and, consequently, the development of the child living in extreme poverty [9].

There is, however, growing evidence that early interventions can prevent developmental loss [5]. For instance, a study comprising more than 127,000 families in 28 developing countries [10] have confirmed an improvement of cognitive and social-emotional development in children under five through enriching caregiving practices. Responsive stimulation delivered at home improved child development and care even after the intervention ended [11, 12]. Such interventions are effective, especially, when they are of higher quality, greater intensity, longer duration, organized at home, and involve the parents [13–16]. The best results are obtained when families have opportunities to practice and receive feedback on the interactions with their children from trained childcare workers [3, 5, 17–20]. Furthermore, home-based stimulation, particularly when mediated by the mothers, shows a sustained positive influence on children’s school attainment, academic performances, vocabulary scores, attitudes towards school and improved social adjustment [19].

In a cluster randomized controlled trial in Colombia, psychosocial stimulation provided at home with play demonstrations on a weekly basis to children aged 12–24 months significantly improved their cognitive and receptive language [15]. Home-based early child development intervention also improved the developmental outcomes of Peruvian children of 6–35 months in age [21]. The 20 years Jamaican follow-up study revealed that, in disadvantaged settings, simple and very early psychosocial stimulation during childhood can have a substantial effect on labor market outcomes and reduce inequality later in life [14]. The returns of early interventions for young children are high during their adult life. Failure to invest early can lead to irreversible damage to children [22].

Early childhood interventions conducted so far have revealed important pieces of evidence. However, studies into the effects of play-assisted stimulation on the overall development of children living with foster families in extreme poverty have, to our knowledge, not been carried out.

In 2013, we assessed the developmental and nutritional status of 819 children under five years old in extreme poverty, and 62 children under six years old in the SOS village in the vicinity of Jimma (Ethiopia). Children in both groups showed developmental problems, particularly in social-emotional and language skills; about 40% of these children were also stunted (submitted for publication). If the poorest and most marginalized children and families are supported early in life with appropriate interventions, the cycle of poverty may be interrupted; sustainable development may be ensured, and child developmental outcomes may be improved [14, 22–26]. If the interventions receive recognition as core strategies for poverty reduction and high returns, using these contributions as inputs to global policy priorities, better outcomes can be achieved [4, 14, 26–29].

With this background, the main objective of this study was to investigate the effect of an intensive home-based play-assisted stimulation integrated into the basic services (a family home, food, clothing, health care, protection and education), provided by SOS village, on the developmental performances of children living with foster families in extreme poverty. The basic services were given to both groups, whereas play-assisted stimulation was not given to the control group. Provision of the basic services and stimulation started simultaneously. It was hypothesized that play-assisted stimulation would improve mainly social-emotional and language skills of children in the intervention group.

Play-assisted stimulation refers to play activities and games for developmental stimulation of children in the intervention group. Clinical nurses (trained as play leaders) taught foster mothers parenting skills, how to interact and play with their children. For each child, the nurses applied these skills for six months, focusing particularly on social-emotional and language development of the children. For the stimulation, they used age- and culture-appropriate play materials and games. The weekly play sessions emphasized improving child-mother interactions and transferring key play skills to sustain these skills. Detailed information about this topic is presented under “design and intervention”. Each child was assessed three times during the study period: at baseline, midline and endline.

## Methods

### Study setting and participants

This study was conducted in Jimma town, South West Ethiopia, with an estimated population of 198, 228 [30]. Amharic and Afan Oromo languages are predominantly spoken in the area.

Participants of the study were children in a foster care program in this extremely poor community, arranged by SOS Children’s Village, and their foster mothers. Extreme poverty is defined as living below the international poverty line of 1.90 USD per person per day [1]. SOS Village provides basic services such as a family home, food, clothing, health care, protection and education for these children. For the foster mothers, they regularly organize training on holistic child development, parenting and care. Based on their willingness and capabilities, the foster mothers were selected by the Women’s and Children’s Affairs Office and SOS Village from among local residents. Children were eligible for the study if they lived in Jimma town, were selected for the foster care program, and their ages were between 3 and 59 months. Children were excluded if they were completely blind or deaf or both, lived outside Jimma town, or had profound intellectual disabilities. This study started in October 2015 and was completed in July 2016.

Most children living with foster families in Jimma are orphaned (lost one or both parents) or abandoned. Unlike children living with their biological families, these foster children may struggle with negative past experiences, and adjustment and attachment problems. These problems could in turn negatively influence their development and behavior [31–34]. This may add more pressure for foster mothers and make parenting a challenging task for them. To minimize the impacts and to accommodate the needs of foster children, the SOS Village in Jimma arranges adequate training for foster mothers and closely supervises their caring practices.

SOS Children’s Village is a family-oriented, independent non-governmental organization working in the spirit of the United Nations Convention on the Rights of the Child. The organization targets children who are orphaned, abandoned or lack care of a family. It has more than 500 villages in 133 countries across the world [35]. The SOS Children’s Village of Jimma in South West Ethiopia was opened in 2012. It offers care in 15 family houses to 150 children under the age of 14 years. In each house, there is one SOS mother and an aunt, offering care for 10 children. As an alternative child care, the village started foster care in October 2015.

### Sample size estimation

A total of 78 children were randomized to the intervention (*n* = 39) and the control (*n* = 39) groups. This sample size was needed to obtain 80% power for detection of a difference of 9% or 0.09 (SD = 0.13) in developmental performance score between the two groups. Calculation to power the study was based on the estimates of the variance in developmental performance ratio scores of 62 children (32 boys and 30 girls) in the SOS Village of Jimma. Their age ranged from 3.5 to 71.8 months [44.6 (21.3) months]. We used the data of SOS children for the power calculation because they had similar characteristics to the children in the intervention study. A 95% level of confidence and two-tailed test was used. This sample size estimate also considered 20% attrition.

### Design and intervention

A randomized single blind controlled trial (parallel) design was used. The random assignment of the children to intervention and control groups was accomplished using computer-generated codes at a 1:1 ratio. The enrollment and allocation of participants was done by an experienced assistant study coordinator. The investigator and those assessing the outcomes were blinded to group assignment. Children in the intervention group received home-based play-assisted stimulation in addition to the basic services provided to children in both groups.

The stimulation activities were carried out by experienced clinical nurses at the children’s home, in cooperation with the foster mothers and other children at home or in the neighborhood. The nurses were intensively trained for more than a month on child development, safety and care. They were also trained on key play principles such as safety, enjoyment and stimulation [36], and effective communication with children and mothers in the context of extreme poverty. Immediately after finishing the theoretical training, they practiced with children and mothers in a similar setting. During all their practical sessions, they were strictly supervised and given feedback to help them master the skills required for the actual intervention works.

The intervention was given during a weekly home visit for 6 months. At every visit, play materials were brought to the home and left for the mother and the child to use. The intervention focused on activities to promote developmental skills and emphasized direct mother-child interactions. Mothers were regularly reminded and motivated to continue practicing the activities and cultural games learned during the home visits.

Fortunately, no visit was cancelled or missed and each intervention child received 24 stimulation sessions. The play materials used to assist the developmental stimulation included culturally appropriate and child friendly dolls, toys, puppets, picture books, card games, cognitive games, drawings, color pencils and papers, simple and playful musical instruments, bells, balls and blocks. Cultural play and games were also used based on the age level of a child. During every home visit, the nurses played with the children (including mothers), progressively trained the mothers, and transferred play skills. These approaches had worked well in previous studies [14, 15, 37, 38]. One home visit session took 60 min on average and the intervention study lasted for six months.

### Outcomes, measurements and instruments

#### Developmental performance

Personal-social, language, fine and gross motor performances of the children were assessed using the culturally adapted and standardized developmental screening tool, the Denver II-Jimma [39]. It has an excellent inter-rater and test-retest reliability [39]. The test took 20 min on average per child. The developmental performance score is defined as the number of age-appropriate test items of a domain a child has successfully passed. On the other hand, social-emotional performance (self-regulation, adaptive functioning, affect, compliance, autonomy, interaction with people and communication behaviors) was assessed using the adapted versions of Ages and Stages Questionnaires: Social-Emotional/ASQ: SE [40, 41]. This developmental screening tool was developed to identify children’s social and emotional competences [41] and is believed to have a high rate of detection for social-emotional problems among young children [32]. An ASQ: SE questionnaire took about 10–15 min to complete for a caregiver. A child’s total social-emotional performance score is obtained by adding the points of all items on a questionnaire. A higher total score shows more social-emotional problems.

#### Anthropometric assessments

Anthropometric assessments were done following the WHO guideline [42]. A child’s weight was measured using a calibrated electronic weighing scale (SECA Uniscale, Hamburg, Germany). For children under two years, length was measured using a length-measuring mat on a flat table (SECA 210, Hamburg, Germany). The height of a child over two years was measured with a Seca 214 Road Rod Portable Stadiometer. The anthropometric measures were converted into Z-scores of Weight-for-Age (WAZ), Height/Length-for-age (HAZ/LAZ), and Weight-for-Height/Length (WHZ/WLZ) using WHO Anthro and Anthro plus software [43].

#### Sociodemographic characteristics

To gather data on the sociodemographic characteristics of the children, their foster mothers and family, a structured questionnaire was used [See Additional file 1]. The variables on which the data were collected were age, sex and birth order of a child, number of peers in the neighbourhood, child-to-child interaction, maternal age, education, occupation, ethnicity and religion, family size and income.

### Assessment procedure

The assessments were made by four trained nurses (assessors). Being blind to the group to which the child belonged, the nurses assessed the children in both groups at the children’s homes. They first administered the structured questionnaire, alongside ASQ: SE; second, the Denver II-Jimma and finally, the anthropometric measurements (weight first, then height/length). The assessment took an hour per child. Each child was assessed three times during the study period: at baseline, midline (after three months) and endline (after six months).

### Statistical analysis

Double data entry was done into EpiData to ensure data quality [44]. For the statistical analysis, SAS Software version 9.4 was used. To compare the intervention and control group at baseline, chi-square tests (χ^2^) were employed for data with categorical outcomes and independent samples t-tests for data with continuous outcomes.

The effects of home-based play-assisted stimulation on the evolution of a child’s developmental performances was investigated by using generalized estimating equations (GEE) for repeated data. Successfully passed test items were used as developmental performances. Analysis of residuals showed a symmetric distribution. The GEE model incorporated two main effects - i.e. a group indicator and a time variable with three time points [baseline, midline (after 3 months) and endline (after 6 months)]- and their interaction term. This model allowed for group-specific evolutions in developmental performance (from baseline-midline and from baseline-endline). An unstructured working correlation matrix was specified for each developmental outcome. Online calculators from Psychometrica were used to obtain effect sizes for the significant intervention effects [45].

Possible intervention effect modifiers were investigated for changes from baseline to endline. Multiple regression models were fitted to the changes from baseline [46]. The model included the group indicator, the covariate/modifier of interest and the interaction term. A significance level of 5% was used and all tests were two-tailed.

## Results

**Fig. 1:**
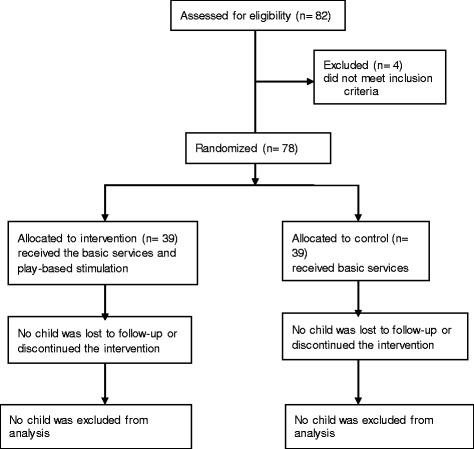
Flow chart from enrollment to data analysis

Initially, 82 children were assessed for eligibility. Four children were then excluded because they did not meet the inclusion criteria. Two children became sick after they had been screened and two other children permanently moved with their families to another place. The remaining 78 children were randomized into intervention and control groups. The intervention children received both the basic services and play-assisted developmental stimulation. Children in the control group received only the basic services. The data of all randomized children in both groups were considered for analyses (Fig. 1). The study was conducted as planned in the original protocol. No harm was inflicted on any of the children in each group as a result of the study.

### Baseline characteristics

Baseline child, maternal and family characteristics of the intervention and control group are presented in Table 1. Significant differences were observed in birth order and maternal age. Fifty-nine percent of the children in the intervention group were born after a third child compared to only 28% in the control group. The majority of foster mothers for children in the control group were younger than those in the intervention group.

**Table 1:** Baseline child, maternal and family variables of the control and intervention groups (*N* = 78)

Variables	Intervention	Control	*p*-value
	(*n* = 39)	(*n* = 39)	
Child variables			
Age (in months)	20.83 (14.76)	21.05 (14.77)	0.949
Birth order (born after third child)	23 (59.00%)	11 (28.20%)	0.006*
Peers in neighborhood (two or more)	34 (87.20%)	34 (87.20%)	1.000
Girls	14 (35.90%)	22 (56.40%)	0.069
Language performance	17.50 (9.00)	17.90 (8.90)	0.860
Personal social performance	14.30 (6.30)	14.40 (6.30)	0.929
Fine motor performance	16.90 (6.20)	16.40 (5.60)	0.747
Gross motor performance	18.70 (7.90)	18.80 (7.30)	0.929
Social-emotional performance	49.00 (17.30)	48.60 (24.50)	0.936
WHZ	0.20 (1.40)	− 0.10 (1.60)	0.490
HAZ	−1.40 (1.50)	−1.20 (1.50)	0.570
WAZ	− 0.60 (1.20)	− 0.70 (1.20)	0.782
Maternal variables			
Age (in years)	40.69 (10.00)	25.87 (5.38)	< 0.001*
Education (Illiterate or ≤ grade 8)	28 (71.80%)	32 (82.10%)	0.282
Occupation (house maid or on street merchant)	30 (76.90%)	35 (89.70%)	0.129
Family variables			
Family size (more than 3)	31 (79.50%)	23 (59.00%)	0.050
Family income (< 1.90 USD/day)	30 (76.90%)	36 (92.30%)	0.060

Chi-square test (n (%) and *p*-value) and independent samples t-test (mean (SD) and *p*-value) were performed for categorical and continuous data respectively

*Indicates significant test

### Effects of play-assisted stimulation on the developmental performances of children

**Fig. 2:**
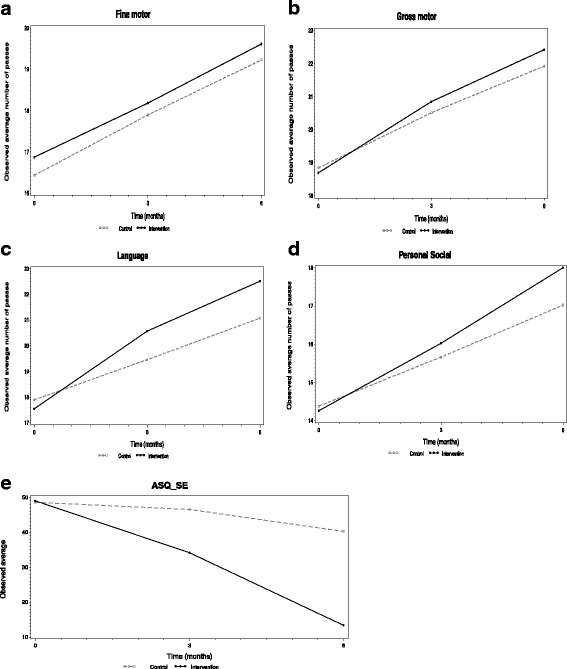
Developmental performances of children in intervention and control (broken line with gray color) group

The observed average evolutions in developmental performance, for the control and the intervention group, are displayed in Fig. 2. For all domains, increases over time were observed.

The statistical analyses, based on generalized estimating equations (GEE), showed that there was a benefit of the intervention for language, social-emotional and personal-social performances (Table 2). For language performances (at midline: β = 1.46, Z = 2.43, *p* = 0.0151, effect size (ES) = 0.34; at endline: β = 1.79, Z = 3.20, *p* = 0.0014, ES = 0.55), children in the intervention group had higher average performance scores than children in the control group. Children in the intervention group also performed better in social-emotional outcome (at midline: β = − 12.73, Z = − 4.07, *p* <  0.0001, ES = − 0.603; at endline: β = − 27.06, Z = − 11.61, *p* <  0.0001, ES = − 1.28). Hence, the benefits of the play-assisted developmental stimulation were already observed after three months for both outcomes. For personal-social (at endline: β = 1.10, Z = 2.63, *p* = 0.0087, ES = 0.56), the beneficial effect of the intervention was significant at endline only (Table 2).

**Table 2:** Intervention effects on the developmental performances of children, using Generalized Estimating Equations (GEE)

	Intervention effect (at 3 months)				Intervention effect (at 6 months)			
Developmental Performances	Estimates(SE)	Z	*P*-value	Effect size	Estimates(SE)	*Z*	*P*-value	Effect size
Language	1.46 (0.60)	2.43	0.0151	0.34	1.79 (0.56)	3.20	0.0014	0.55
Personal Social	0.49 (0.37)	1.31	0.1907	–	1.10 (0.42)	2.63	0.0087	0.56
Social-Emotional	−12.73 (3.13)	− 4.07	< 0.0001	− 0.603	−27.06 (2.33)	−11.61	< 0.0001	−1.28
Fine Motor	− 0.14 (0.40)	− 0.36	0.7177	–	− 0.02 (0.45)	− 0.05	0.9562	–
Gross Motor	0.48 (0.57)	0.85	0.3938	–	0.63 (0.58)	1.09	0.2750	–

Intervention effect-the difference in developmental performance between intervention and control groups at 3 or 6 months

*SE* Standard Error, *Z* test statistic for GEE Models, *P*-value significance level

### Dependency of intervention effect on baseline characteristics

For the changes in developmental performance (endline versus baseline), we examined the possible effects of baseline variables (child’s age, sex, birth order, developmental performance, WHZ, WAZ, HAZ, child-to-child interaction, maternal age and education, family size and income) on the intervention effect. The magnitude of the intervention effect for language depended on the child’s sex; for personal-social on family income; for fine motor skills on WHZ, WAZ, child-to-child interaction and maternal education; and for gross motor skills on WAZ and maternal education. For social-emotional performance, no dependency was observed (Table 3). Regarding language performance, the intervention at endline was more effective for boys than for girls. For personal-social performance, the intervention was more effective for children in the intervention group whose families’ monthly income was lower.

**Table 3:** Dependency of intervention effect on baseline variables based on multiple regression analysis

	Developmental performances									
	LA		PS		SE		FM		GM	
Baseline variables	β	*P*	β	*P*	β	*P*	β	*P*	β	*P*
Child’s age	0.01	0.89	− 0.03	0.29	− 0.35	0.25	− 0.02	0.60	0.04	0.38
Birth order	0.02	0.94	− 0.04	0.87	1.25	0.56	− 0.02	0.92	0.17	0.57
Family size	0.29	0.44	− 0.41	0.18	1.87	0.53	− 0.19	0.58	− 0.40	0.35
Income	0.00	0.84	− 0.002	0.03*	0.00	0.99	0.00	0.50	0.00	0.99
WHZ	− 0.11	0.78	0.24	0.43	−1.52	0.61	0.99	0.01*	0.65	0.11
WAZ	0.03	0.96	0.65	0.08	1.20	0.75	1.50	0.01*	1.33	0.01*
HAZ	0.11	0.78	0.43	0.17	2.81	0.35	0.39	0.25	0.66	0.13
Performance	− 0.01	0.84	− 0.02	0.74	0.05	0.65	− 0.04	0.53	0.08	0.22
Maternal age	0.04	0.61	0.02	0.81	− 0.28	0.68	0.06	0.60	− 0.01	0.96
Maternal education	− 1.07	0.78	− 2.87	0.34	− 0.67	0.98	− 7.40	0.02*	− 8.83	0.03*
Child-child interaction	− 5.54	0.09	1.08	0.69	5.55	0.83	− 7.80	0.01*	−1.71	0.64
Child’s sex	3.03	0.01*	0.28	0.76	− 2.24	0.81	1.06	0.32	0.18	0.89

*β* regression coefficient, *P* (*P*-value) significance level, *LA* language, *PS* personal social, *SE* social-emotional, *FM* fine motor, *GM* gross motor, *WHZ* weight-for-height/length z score, *WAZ* weight-for-age z score, *HAZ* height/length-for-age z score;

*Indicates significant test

## Discussion

Play-assisted stimulation integrated into basic services and given at home on weekly basis significantly improved the social-emotional, language and personal-social performances of children living with their foster families in the context of extreme poverty. At midline, we detected improvements in social-emotional and language outcomes for the intervention group. For personal-social, significant improvements were observed only at the endline.

Though the intervention effects were not observed as early as in this study, other intervention studies have also confirmed that intensive home visits can improve children’s developmental outcomes [14, 15, 21, 38, 47, 48]. Interventions on maternal play and parenting skills have also improved young children’s social, emotional, communication, language and cognitive competence besides improving maternal warmth and cognitively responsive behavior [18, 49–53].

The effect sizes for the significant developmental performances in this study range from medium to large [54]. The intervention effect in this study has shown clinical relevance, especially for social-emotional and language performances. For the social-emotional skills, on average, after three months of intensive intervention, about 13 total scores of social-emotional problems were reduced, and after six months, 27 total scores were abridged for the children in the intervention group. For language skills, 1.5 items at three months and 1.8 items at six months were improved. Assuming that these improvement rates will be sustained, because of the skills transferred to and mastered by mothers during the intervention, children in the intervention group may further improve their social-emotional and language development. At the end of the study, most mothers also pointed out that they had observed encouraging developmental improvements in their children. They also found the play and parenting skills highly relevant to make the observed developmental performance changes sustainable.

There was no significant benefit of the intervention on fine and gross motor performances during the six months follow-up. This may be because the basic services benefited children in both groups in their motor development, and the intervention did not add extra value for children in the intervention group. Another explanation could be that the intervention cannot improve motor development within a period of only six months. Previous studies have also revealed non-significant effects of short-term developmental interventions on motor performance [15, 55, 56]. In a randomized play-based home intervention for under-25 month age children in low socioeconomic families, the effects on motor development were observed more than one-year after the intervention ended [57]. A similar study on children of 24 months in age showed improvement two years after the end of the intervention [11]. Furthermore, a home visiting early child development (ECD) program in the Caribbean significantly improved fine motor skills of birth to 3 year-old children, one year after the implementation of the program [58].

We investigated effects of baseline variables on the magnitude of the intervention effect and observed that families with lower income benefited more from the intervention. Most of the children from lower income families have less infrastructure, interaction time and opportunities for stimulation. As a result, they may be more delayed developmentally. Because of their lower baseline developmental level, the intervention effect might be more pronounced when they are given additional stimulation compared to those with a better income and a better chance of getting home-based stimulation. Evidence of this kind has already been documented in countries such as Jamaica, Colombia and Peru [14, 15, 21]. What is not yet clear is that the average language performance for the boys in the intervention group is higher than that of the girls. This may partly be because of a deep-rooted cultural practice and bias towards being a boy or a girl. In Ethiopia or other African countries, family members (particularly mothers) show more preference to, give attention to, talk to and interact more with boys than girls. This maternal behavior could result in language skill differences between the two sexes. Moreover, no baseline age dependency of intervention effect for any of the developmental outcomes was observed. This implies that both younger and older children benefited from the intervention in the same manner.

This study can be scaled-up in low-resource settings and home environments. It is feasible and cost-effective. For example, the intervention cost per child for six months was only 35 USD. In a home setting, the play activities can easily be integrated into the day-to-day activities of mothers and children. The play materials are also of low cost and locally available. Age-appropriate cultural games can be used effectively. Because both the children and mothers enjoy the play and interaction sessions, the one-hour weekly home visit is appropriate. Moreover, the skills are easily transferable and sustainable.

The major limitation of this study is its short period of follow-up. The study was planned this way mainly because of financial constraints. Fortunately, it was observed that the houses of families of children in the intervention and control groups were fairly far away from each other, i.e. the possibilities of sharing information and intervention materials were minimal. Nonetheless, there might be accidental contamination. The use of the developmental screening tool, Denver II, could also be a limitation for its limited specificity [59]. However, we adapted and standardized the test and used a continuous scoring system to overcome possible limitations.

If Western developmental assessment tools were used in different cultural contexts such as low-income and middle-income countries without adapting and standardizing, the developmental outcomes would not be valid and dependable [60–62]. In an attempt to minimize most of the limitations, we used culturally appropriate tools. Regarding Denver II-Jimma, among 125 Denver II test items, 55 (20 personal-social, 18 fine-motor, 15 language and 2 gross-motor) were theoretically identified as culture-specific. These 55 items were piloted through exploratory surveys and discussed at a consensus meeting. Only 36 of them needed adaptation. The other 19 items were retained. Adaptation, re-adaptation and further fine-tuning of Denver II test items resulted in the Denver II-Jimma, which comprises 36 adapted and 89 original Denver II items. Inter-rater reliability of Denver II-Jimma was excellent (kappa > 0.83) for all tested items [39].

## Conclusion

In conclusion, if a quality and intensive home-based play-assisted stimulation is given in a resource-limited context, the benefits for children under five are quick and meaningful, particularly for social-emotional and language skills. The sustainability of the benefits of the family-involving and skill-transferring intervention study can be high. Future research should focus on longer duration of intervention, to observe improvements in all the developmental performances of children.

## Additional file

Additional file 1: Sociodemographic Questionnaire. (DOC 79 kb)

## Abbreviations

HAZ: Z-score of Height-for-Age
LAZ: Z-score of Length-for-Age
WAZ: Z-score Weight-for-Age
WHZ: Z-score of Weight-for-Height
WLZ: Z-score of Weight-for-Length
