# Supplementary material for: Effects of home-based play-assisted stimulation on developmental performances of children living in extreme poverty: a randomized single-blind controlled trial
Source: BMC Pediatr. 2018 Feb 5;18:29. doi: 10.1186/s12887-018-1023-0 (PMC5800292; doi:10.1186/s12887-018-1023-0)
Supplement: Additional file 1: — Sociodemographic Questionnaire. (DOC 79 kb) [file 12887_2018_1023_MOESM1_ESM.doc]

Date of today: ------------------------------- Code for respondent/caregiver ----------------------------

Residential address: woreda/district ---------------------------------------Kebele--------------------------------Garee--------------------

Gooti: ----------------------------------- House Number---------------- Special name of the place: --------------------------------------------

|  |  | A | B | C | D | E | F. | G | H |
| --- | --- | --- | --- | --- | --- | --- | --- | --- | --- |
| 1 | Age of a mother: -------------- | less than 20 yr | 20-30 ¯yr | 31-40 yr | *above 41 yrs* |  |  |  |  |
| 2 | Mother’s religion | Islam | Orthodox Christian | protestant christian | traditional religion | Catholic | other |  |  |
| 3 | Education level of a mother | illiterate | grade 1-8 | grade 9-12 | certificate | diploma | degree & above |  |  |
| 4 | Occupation of a mother  ------------------------ | house wife | teacher | office worker | On street merchant | Field work | Traditional farm | Trade | other |
| 5 | Ethnicity of a mother  ----------------------------- | Oromo  Amhara | Tigre  Gurage | Silte | Dawuro | Yem | Keficho | Welayita | other |
| 6 | Monthly income of a family  -------------------------------- | less than 500 birr | 500-1500 birr | 1501-3500 birr | above 3500 |  |  |  |  |
| 7 | Age of a child: ------------------ |  |  |  |  |  |  |  |  |
| 8 | Sex of a child | Girl | Boy |  |  |  |  |  |  |
| 9 | *Birth order of the child* | first born | 2^nd^ born | *3^rd^ born* | *between 3^rd^ and last born child* | *last born* |  |  |  |
| 10 | *Family size*  *------------------------------------* | *less than 3* | *3-6* | *7-10* | *above 10* |  |  |  |  |
| 11 | *Number of children the child meets in neighbor: ---------------* | *less than 3* | *3-6* | *7-10* | *above 10* |  |  |  |  |
| 12 | *Frequency of the child’s interaction with other children* | always | sometimes | *never interacts with others* |  |  |  |  |  |
